# Effectiveness of Risdiplam Treatment in Adult Patients With Spinal Muscular Atrophy Type IIb–III

**DOI:** 10.31083/RN44408

**Published:** 2026-01-26

**Authors:** Daniel Apolinar García Estévez

**Affiliations:** ^1^Neurology Service, Ourense University Hospital, 32005 Ourense, Spain; ^2^Clinical Neuroscience Group, Galicia Sur Health Research Institute (IIS Galicia Sur), Alvaro Cunqueiro Hospital, 36123 Vigo, Spain

**Keywords:** spinal muscular atrophy, risdiplam, neurofilaments, motor neurone disease, glial fibrillary acidic protein, biomarkers

## Abstract

**Introduction:**

Risdiplam is a pharmacological agent developed for the treatment of spinal muscular atrophy (SMA) associated with 5q deletion, with the therapeutic objective of increasing the concentration of the survival motor neuron 2 protein. Most clinical trials and real-world studies have focused on pediatric and young adult populations. Our aim was to assess the effectiveness of risdiplam treatment in adult patients with SMA type IIb and III.

**Methods:**

We studied 8 adult patients with SMA (3 females/5 males). Patient functionality was assessed using the Egen Klassifikation version 2 (EK2) scale, upper limb function with the 9-hole peg test (9HPT, seconds), and respiratory function with peak flow (L/min) and sniff nasal inspiratory pressure (SNIP, cmH_2_O). Plasma levels of neurofilament light chain (NFL, pg/mL) and glial fibrillary acidic protein (GFAP, pg/mL) were also measured. Patients were evaluated at baseline, and after 6 and 12 months of treatment.

**Results:**

The median age was 55 years (range: 41–66). At 12 months, EK2 scores showed a trend toward improvement in swallowing [item 16] (*p* = 0.06), peak flow increased significantly (244 ± 112 vs. 259 ± 124 L/min, *p* = 0.036), and there was a trend toward decreased NFL levels (11.4 ± 4.9 vs. 9.4 ± 2.7 pg/mL, *p* = 0.093). Both NFL and GFAP concentrations were negatively correlated with peak flow and SNIP values.

**Conclusions:**

In our series, treatment with risdiplam may stabilize adult patients with type IIb–III SMA.

## 1. Introduction

Spinal muscular atrophy (SMA) is a neurodegenerative and genetically determined disease that affects motor neurons in the spinal cord, presenting with muscle atrophy and weakness and associated with difficulties in feeding and breathing [1, 2]. Genetically, in 95% of cases SMA is caused by a homozygous deletion in the survival motor neuron 1 (*SMN1*) gene located on the short arm of chromosome 5, and the remaining cases result from a point mutation in the *SMN1* gene, which causes a decrease in the SMN (Survival Motor Neuron) protein. The different phenotypes of SMA patients are related to both the age of onset of symptoms and the number of copies of the *SMN2* paralogue gene [3, 4], although this fact alone is not sufficient to explain inter- and intra-individual variability [5].

In recent years, there has been a shift from recommending supportive interventions to patients to utilizing currently available pharmacological treatments that increase the concentration of functional SMN protein, which modifies the course of the disease. These treatments are nusinersen (antisense oligonucleotide), risdiplam (a modifier of SMN2 pre-mRNA splicing to include exon 7), and gene therapy with onasemnogene abeparvovec (replacement of the *SMN1* gene via the Adeno-associated virus serotype 9 (AAV9) viral vector) [1, 2, 3].

SMA is one of the most common autosomal recessive diseases in children, with an incidence of 1 case per 5000–10,000 live births and a deletion carrier frequency in the general population of approximately 1 case per 50–100 inhabitants [1, 2]. Based on this epidemiological reality, most of the studies evaluated by drug and medical device regulatory agencies for the approval of different therapies that modify the course of the disease mainly included pediatric patients or young adults [1, 3]. Therefore, experience with adult patients must be acquired progressively based on real-life studies as these patients are included in follow-up studies.

Adult patients over the age of 40 are underrepresented in clinical trials of risdiplam [6], and therefore the therapeutic and neuroprotective effects of risdiplam in this age group should be monitored with particular interest. In this regard, in this article we present our experience with risdiplam treatment in a series of adult patients with SMA types IIb–III who were followed clinically and biochemically for 12 months.

## 2. Patients and Methods

The study design is descriptive, observational, and prospective, and it was carried out at the Neuromuscular Clinic of our Neurology Department. Adult patients diagnosed with SMA type IIb–III with genetic confirmation of the 5q deletion in the *SMN1* gene (n = 8) were studied. Two patients who were receiving treatment with nusinersen (follow-up of 26 and 22 months, respectively) continued treatment of the disease with Risdiplam, which was started 4 months after the last intrathecal infusion of nusinersen. Only one patient was ambulatory, and the rest were sitters.

**Fig. 1.**
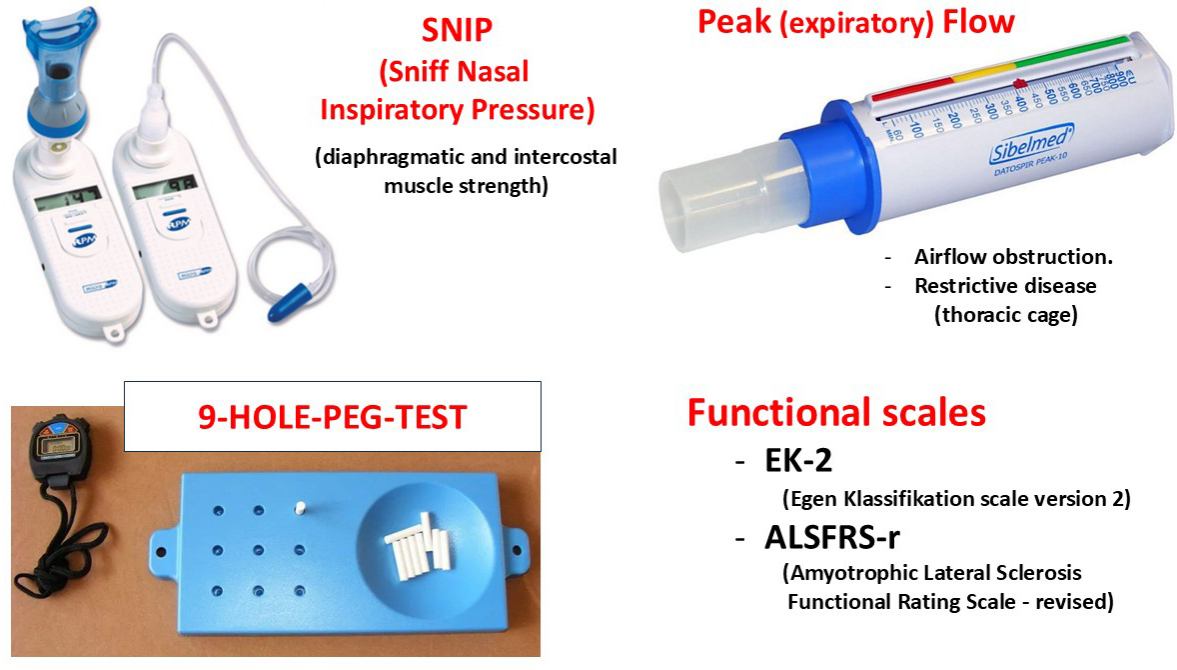
**Materials and methods**. Peak flow and SNIP techniques were used to assess respiratory function, and the 9-Hole Peg Test was used to assess upper extremity function. SNIP stands for sniff nasal inspiratory pressure.

The management of periodic assessments of these patients is complex. Seven of the patients are non-ambulatory and reside in locations more than 60 km from the referral hospital, and thus require adapted medical transportation to attend the various specialist consultations. In addition, most of these patients have significant difficulties in correctly performing a formal respiratory function study with spirometry tests. Another barrier to comprehensive care for our adult SMA patients in our healthcare area is that there is currently no easy access to professionals trained in the administration of the various motor scales commonly used in the clinical follow-up of this pathology, such as the RULM (Revised Upper Limb Module), the Hammersmith Functional Motor Scale Expanded (HFMSE), and the MFM-32 (32-item Motor Function Measurement) scale. For these reasons, and due to time constraints in outpatient consultations, the protocol for monitoring adult patients with SMA in our clinic consists of the following processes: (1) functional assessment of patients is performed using the Egen Klassifikation Scale version 2 (EK2) and the ALSFRS-r (Amyotrophic Lateral Sclerosis Functional Rating Scale - revised) scales; (2) respiratory function is quantified by measuring peak flow (PF, L/min) [7, 8, 9, 10] and SNIP (Sniff Nasal Inspiratory Pressure, cmH_2_O) [11, 12, 13, 14, 15]; and (3) upper extremity function (hand and fingers) is assessed using the 9-Hole Peg Test (9HPT), which is the test commonly used in multiple sclerosis clinics to assess the progression of upper extremity disability in these patients [16, 17, 18]. In the 9HPT, the patient is asked to insert and then remove the nine pegs from the holes in the board, one by one, using only the hand being assessed, and the time is measured in seconds (Fig. 1). Peak flow allows us to assess bronchial obstruction and restrictive chest wall pathology, and is a measure that approximates the quantification of maximum expiratory pressure; in practice, five measurements are taken and both the maximum value (maximum PF) and the average of the five measurements (mean PF) are recorded. SNIP is a measure of the strength of the diaphragmatic and intercostal muscles and is an approximation of the maximum inspiratory pressure value. The SNIP measurement process consists of placing a measuring probe in the nostril through which the patient breathes in most effectively, and having the patient take 10–15 rapid and vigorous breaths, with the highest measurement being reflected on the display.

Plasma samples (EDTA tubes) were collected from all patients to determine neurofilament light chain (NFL) and glial fibrillary acidic protein (GFAP) levels. Samples were collected in the week prior to the start of risdiplam treatment (baseline sample) and at 12 months of follow-up, and following centrifugation (10 minutes, 40 rpm), the plasma was aliquoted and stored at –80 °C. Plasma NFL (pg/mL) and GFAP (pg/mL) levels were determined at the Bellvitge Biomedical Research Institute (IDIBELL, L´Hospitalet de Llobregat, Barcelona, Spain) using the SIMOA technique.

This study has been approved by the Galician Drug Research Ethics Committee (Registration Code: 2024/142). Patients gave their informed consent to participate in the study.

## 3. Statistical Analysis

Statistical analysis was performed using IBM SPSS Statistics software, version 29.0 (IBM Corporation, Armonk, NY, USA). Nonparametric statistics were used. The comparison between the groups of related variables (pre-treatment and post-treatment at 6 and 12 months) was performed using the Friedman test, and in the event of statistically significant differences, comparisons between groups were performed using the Wilcoxon test. Correlations were performed using Spearman’s rho. Statistical significance was considered to be *p* < 0.05.

## 4. Results

The baseline characteristics of the patients are shown in Table 1. Treatment with risdiplam was well tolerated, except for by one patient who experienced gastric discomfort throughout the observation period (12 months), which did not result in discontinuation of the medication due to adverse effects.

**Table 1.** **Descriptive characteristics of the patients**.

	Patient 1	Patient 2	Patient 3	Patient 4	Patient 5	Patient 6	Patient 7	Patient 8
Sex (M/F)	M	M	M	M	F	F	F	M
Age (years)	66	41	63	41	57	51	60	53
*SMN1* 5q deletion	Yes	Yes	Yes	Yes	Yes	Yes	Yes	Yes
*SMN2* (number of copies)	4	4	3	4	3	4	4	3
Severe scoliosis	No	No	Yes	Yes	Yes	No	No	No
Phenotype	Sitter	Sitter	Sitter	Sitter	Sitter	Sitter	Sitter	Walker
Previous nusinersen	Yes (26 months)	Yes (22 months)	No	No	No	No	No	No

M, Male; F, Female; *SMN*, survival motor neuron.

**Fig. 2.**
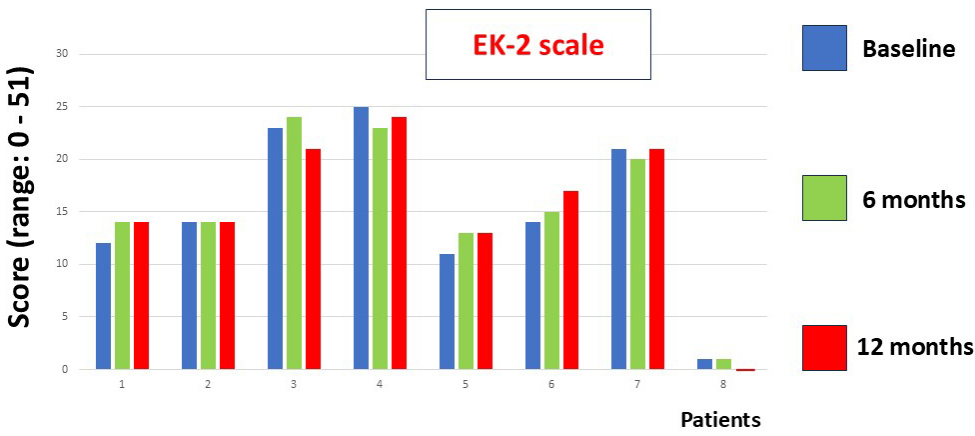
**This figure shows the EK2 scale scores at baseline and at 6 and 12 months of follow-up**. No statistically significant differences were found (Friedman test).

**Fig. 3.**
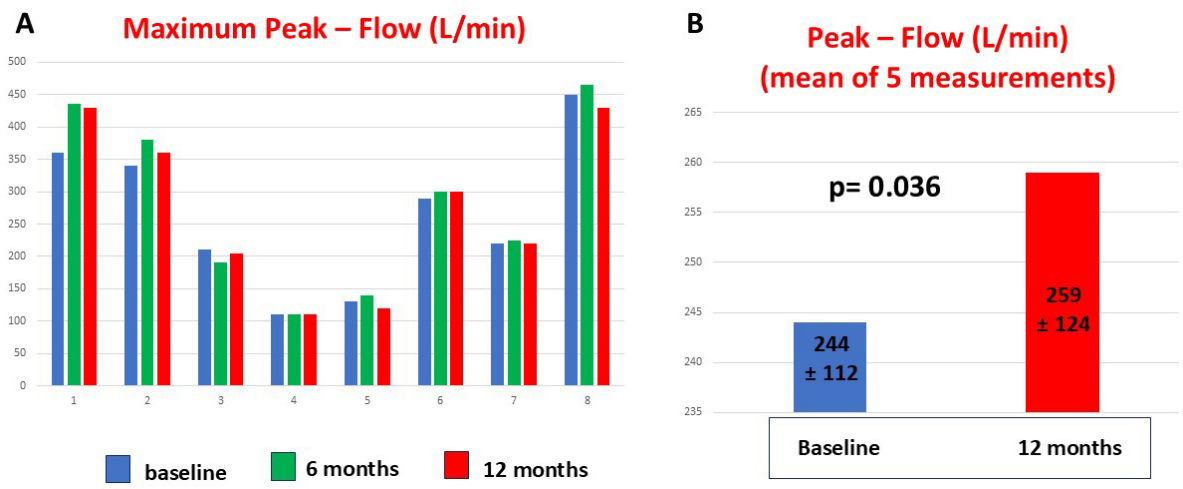
**This figure shows the maximum peak flow values of adult patients with SMA obtained at baseline and at 6 and 12 months of follow-up**. Patients 3, 4, and 5 had severe scoliosis (A). Patients showed an improvement in mean peak flow value at 12 months after starting treatment (B).

In terms of the variables studied, we found no statistically significant differences at 6 months or 12 months of follow-up (Table 2). Although there were no changes in the functional assessment scales (EK2, ALSFRS-r), on the EK2 scale, item 16 (swallowing) tended to improve at 12 months of follow-up, with 4 patients decreasing by 1 point while the other 4 remained stable with a score of zero (*p* = 0.06) (Fig. 2). Respiratory function measured with peak flow and SNIP remained stable throughout the treatment. However, mean PF showed a statistically significant increase from baseline at the 12-month follow-up (244 ± 112 vs. 259 ± 124 L/min, *p* = 0.036) (Fig. 3). The 9HPT showed no significant differences for dominant or non-dominant hand function.

**Table 2.** **Functional scale values at the different visits**.

	Baseline visit	6-month visit	12-month visit	*p*
EK2 scale	15.1 ± 7.8	15.5 ± 7.3	15.4 ± 7.5	0.895
ALSFRS-r scale	29.6 ± 6.3	30.5 ± 7.3	30.7 ± 6.6	0.147
9-HPT [dominant hand] (seconds)	31.0 ± 3.7	30.3 ± 2.4	33.2 ± 9.7	0.957
9-HPT [non-dominant hand] (seconds)	43.5 ± 10.7	44.4 ± 11.4	42.5 ± 9.8	0.846
Peak flow (L/min)	264 ± 118	281 ± 135	272 ± 128	0.096
SNIP (cmH_2_O)	72 ± 24	76 ± 30	73 ± 29	0.657

Comparisons were made using the Friedman test; EK2, Egen Klassifikation version 2; 9-HPT, 9-Hole Peg Test.

**Fig. 4.**
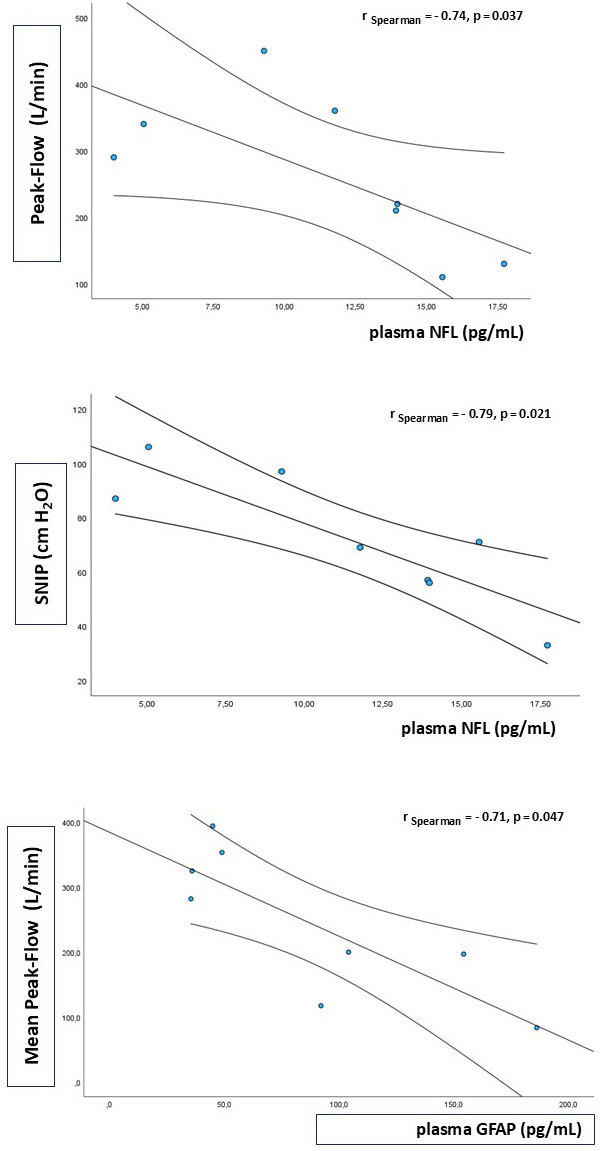
**Biomarkers and lung function**. The upper image (peak flow) and the middle image (SNIP) show the negative correlations between baseline plasma NFLs and respiratory function measurements. In the bottom image, baseline plasma GFAP levels were negatively correlated with respiratory function (mean peak flow).

Plasma GFAP levels did not change after 12 months of treatment with risdiplam (97 ± 55 vs. 96 ± 49 pg/mL, *p* = 0.779), and although there was a decrease in plasma NFL concentration, it did not reach statistical significance (11.4 ± 4.9 vs. 9.4 ± 2.7 pg/mL, *p* = 0.093). The three patients with 3 copies of SMN2 had a higher mean plasma NFL concentration (13.64 ± 4.23 pg/mL) than patients with 4 copies of SMN2 (10.06 ± 5.24 pg/mL). NFL levels were negatively correlated with the maximum PF value (r = –0.74, *p* = 0.037), the mean PF (r = –0.76, *p* = 0.028) and the SNIP (r = –0.79, *p* = 0.021); and GFAP correlated negatively with the mean PF value (r = –0.71, *p* = 0.047) (Fig. 4). The three patients with an EK2 scale score of greater than 20 points had the highest GFAP levels (148 ± 41 pg/mL) compared to patients with a lower score (66 ± 37 pg/mL). One patient reported a worsening of her clinical condition after starting treatment with risdiplam, reflected by an increase in her EK2 scale score from a baseline value of 14 to a value of 17 at 12 months of follow-up, mainly due to a loss of upper extremity function, which resulted in a 6-second increase on the 9HPT test. Plasma biomarkers showed an increase in GFAP from 35 to 80 pg/mL and in NFL from 4 to 8 pg/mL. This patient discontinued risdiplam treatment at 12 months of follow-up.

## 5. Discussion

Risdiplam is an SMN2 pre-mRNA splicing modifier that shifts splicing in the *SMN2* gene to include the deleted exon 7 in the mRNA transcript, resulting in increased expression of functional and stable SMN protein [4].

It is difficult to compare our results with other clinical studies of adult patients with SMA because, although some have included patients up to 60 years of age, they have not compared them with young patients, nor have they used the same assessment scales as in our study [19]. What does seem to have been demonstrated is that risdiplam is a safe, effective treatment that stabilizes or improves patients with SMA (types I, II and III), and that its effect is greater in the presymptomatic and early stages of the disease; furthermore, it has been suggested that risdiplam may not have a favorable effect on respiratory function, which is attributed to chest wall deformities [6, 19].

In adult patients with SMA, treatment with nusinersen or risdiplam aims to slow the progression of the disease and to afford patients stability in their clinical condition [20]. In this context, in our series of patients, we have observed stability in functional assessment scales (EK2 and ALSFRS-r) and in manual dexterity with the 9HPT. However, as a group, our patients treated with risdiplam showed significant improvements in swallowing (item 16 on the EK2 scale) and respiratory function (peak flow). Individually, patients described very heterogeneous impacts of the treatment, with some reporting improvements in head control, in swallowing, in the sensation of more easily filling their lungs with air, in the ease of moving their forearm from a pendulum position to the armrest, greater ease in flexing their forearm and bringing their hand to their mouth, or a more effective cough to clear secretions.

The 9HPT found that upper extremity function worsened in two patients, with patient 1 reporting greater weakness in wrist extension (an action required to perform the 9HPT) in the three months prior to the 12-month assessment, and patient 6 reporting that, despite starting treatment with risdiplam, her previous clinical condition of progressive loss of upper extremity function continued during the months of treatment. This patient is the one mentioned previously in the results section, who ultimately discontinued treatment with risdiplam at 12 months of follow-up.

In healthy subjects, the serum NFL level is less than 10 pg/mL, 25% higher than plasma levels [21, 22]. Our biomarkers are determined in plasma, so the estimated serum NFL level in our series would correspond to a concentration of 14 pg/mL, indicating that the NFL levels of SMA patients in our study are higher than those of healthy controls. NFL levels are also known to be higher in SMA patients with ≤2 copies of *SMN2* compared to those with a higher number of copies, and this finding may influence the absolute value in our patients who have 3 or 4 copies and would therefore express a lower NFL concentration [23]. In our study, the mean concentration of the GFAP and NFL biomarkers did not change significantly with risdiplam treatment, but notably, patients with an EK2 score of ≥20 had the highest GFAP concentrations, suggesting a relationship between this biomarker and patients’ functional status. We also found a negative correlation between peak flow and SNIP values and plasma concentrations of NFL and GFAP, suggesting that these biomarkers may be useful in assessing respiratory function in patients with SMA. We cannot rule out the possibility that these biomarkers may be predictive of the individual SMA patient’s clinical evolution, or of their response to treatment with risdiplam. Along these lines, in the patient whose EK2 scale and 9HPT results worsened, the concentrations of both biomarkers doubled from the start of treatment, and her clinical worsening thus correlated with the increase in plasma biomarkers.

The limitations of our study include the small number of patients included in the follow-up, the lack of a control group, and the fact that 25% of the study population had previously received treatment with nusinersen. In addition, the clinical follow-up period is probably too short to assess the true effectiveness of a treatment that modifies the course of a genetically determined disease.

## 6. Conclusions

In summary, in our series of adult patients with SMA type IIb and III who received treatment with risdiplam, after one year of follow-up, both clinical and biochemical stabilization were observed. Furthermore, our data suggest that as a group, patients treated with risdiplam exhibit a tendency towards improvement in swallowing and respiratory function as assessed by peak flow. Further studies are needed to confirm the usefulness of biomarkers (NFL, GFAP) in the clinical follow-up of patients with SMA.

## Data Availability

The datasets generated and analyzed during the current study are available from the corresponding author on reasonable request.
