# Supplementary material for: Effectiveness of Risdiplam Treatment in Adult Patients With Spinal Muscular Atrophy Type IIb–III
Source: Rev Neurol. 2026 Jan 26;81(1):44408. doi: 10.31083/RN44408 (PMC12873657; doi:10.31083/RN44408)
Supplement: Translation

# Translation: Efectividad del Tratamiento con Risdiplam en Pacientes Adultos con Atrofia Muscular Espinal de Tipo IIb–III

**Published:** 2026-01-26

**Authors:** Daniel Apolinar García Estévez

**Affiliations:** ^3^Servicio de Neurología, Complejo Hospitalario Universitario de Ourense, 32005 Ourense, España; ^4^Grupo de Investigación en Neurociencias Clínicas, Instituto de Investigación Sanitaria Galicia Sur (IIS Galicia Sur), Hospital Álvaro Cunqueiro, 36123 Vigo, España

**Keywords:** atrofia muscular espinal, risdiplam, neurofilamentos, proteína ácida fibrilar glial, enfermedad de neuronamotora, biomarcadores

## Abstract

**Introducción:**

Risdiplam es un fármaco diseñado para el tratamiento de la atrofia muscular espinal (AME) con deleción 5q, y cuya finalidad es incrementar la concentración de la proteína survival motor neuron 2. La mayoría de los ensayos clínicos y de estudios en vida real se realizaron en la población pediátrica y en adultos jóvenes. Nuestro objetivo fue valorar la efectividad del tratamiento con risdiplam en pacientes adultos con AME de tipo IIb y III.

**Métodos:**

Se estudiaron 8 pacientes adultos con AME (3 mujeres/5 hombres). La funcionalidad global de los pacientes se valoró con la escala Egen Klassifikation versión 2 (EK2), la funcionalidad específica de los miembros superiores con el 9-hole-peg test (9HPT, segundos), y la función respiratoria con el peak-flow (L/min) y el sniff nasal inspiratory pressure (SNIP, cmH_2_O). También se determinaron los niveles plasmáticos de neurofilamentos de cadena ligera (NFL, pg/mL) y de la proteína ácida glial fibrilar (GFAP, pg/mL). Los pacientes se valoraron a nivel basal, 6 meses y 12 meses de iniciado el tratamiento.

**Resultados:**

La mediana de edad fue de 55 años (rango: 41–66). A los 12 meses, a nivel de la escala EK2 hubo una tendencia (*p* = 0,06) a la mejoría en la deglución (item 16), el peak-flow se incrementó significativamente (244 ± 112 vs. 259 ± 124, *p* = 0,036), y hubo una tendencia al descenso de los NFL (11,4 ± 4,9 vs. 9,4 ± 2,7, *p* = 0,093). Tanto la concentración de los NFL como de la GFAP se correlacionaron negativamente con el peak-flow y la SNIP.

**Conclusiones:**

En nuestra serie, el tratamiento con risdiplam podría estabilizar a los pacientes adultos con AME de tipo IIb–III.

## 1. Introducción

La atrofia muscular espinal (AME) es una enfermedad neurodegenerativa y genéticamente determinada que afecta a las neuronas motoras de la médula espinal, cursando con atrofia y debilidad muscular, y asocia dificultades para la alimentación y la respiración [1, 2]. Desde un punto de vista genético la AME está causada, en el 95% de los casos, por una deleción homocigótica en el gen survival motor neuron 1 (*SMN1*) situado en el brazo corto del cromosoma 5, y los casos restantes se originan por una mutación puntual en el gen *SMN1*, lo que provoca una disminución de la proteína SMN (del inglés, Survival Motor Neuron). Los diferentes fenotipos de los pacientes con AME están relacionados tanto con la edad de inicio de los síntomas como con el número de copias del gen parólogo* SMN2* [3, 4], aunque este hecho por sí solo no es suficiente para explicar la variabilidad inter e intraindividual [5].

En los últimos años se ha pasado de indicar a los pacientes intervenciones de soporte, a disponer actualmente de tratamientos farmacológicos que permiten incrementar la concentración de proteína SMN funcional, lo que se traduce en una modificación del curso evolutivo de la enfermedad, y estos son el nusinersen (oligonucleótido antisentido), el risdiplam (un modificador del empalme del pre-ARNm de la proteína SMN2 para incluir el exón 7) y la terapia génica con onasemnogene abeparvovec (un reemplazamiento del gen *SMN1* a través del un vector víríco conocido como adeno-associated virus serotype 9 (AAV9)) [1, 2, 3].

La AME es una de las enfermedades con herencia autosómica recesiva más frecuente en la edad pediátrica, con una incidencia de 1 caso por cada 5000–10.000 nacidos vivos, y con una frecuencia de portadores de la deleción en la población general de aproximadamente 1 caso en 50–100 habitantes [1, 2]. En base a esta realidad epidemiológica, la mayoría de los estudios que fueron evaluados por las agencias reguladoras de los medicamentos y productos sanitarios, para la aprobación de las diferentes terapias modificadoras del curso evolutivo de la enfermedad, incluyeron fundamentalmente pacientes en edad pediátrica o eran adultos jóvenes [1, 3]. Por tanto, la experiencia con pacientes adultos se tiene que ir adquiriendo progresivamente en base a los estudios en vida real a medida que se vayan incluyendo estos pacientes en los estudios de seguimiento.

De los ensayos clínicos realizados con risdiplam se observa que los pacientes adultos con edades superiores a los 40 años están escasamente representados [6], y por tanto el efecto terapéutico y neuroprotector del risdiplam en este grupo etario debe estar bajo un seguimiento clínico de especial interés. En este sentido, en este artículo presentamos nuestra experiencia con el tratamiento de risdiplam en una serie de pacientes adultos con AME tipos IIb–III seguidos clínica y bioquimicamente durante 12 meses.

## 2. Pacientes y Métodos

El diseño del estudio es descriptivo, observacional y prospectivo, y se ha llevado a cabo en la consulta de Neuromuscular de nuestro servicio de Neurología. Se han estudiado los pacientes adultos con diagnóstico de AME de tipo IIb–III con confirmación genética de la deleción 5q en el gen *SMN1* (n = 8). Dos pacientes que estaban recibiendo tratamiento con nusinersen (seguimiento de 26 y 22 meses, respectivamente), continuaron el tratamiento de la enfermedad con Risdiplam, que se inició a los 4 meses de la última infusión intratecal de nusinersen. Sólo un paciente era ambulante y los restantes eran fenotipo sitter.

La gestión de la valoración periódica de estos pacientes es compleja. Siete de los pacientes no son ambulantes, y residen en localidades situadas a más de 60 km del hospital de referencia, de forma que precisan disponer de un transporte sociosanitario adaptado para poder acudir a las diferentes consultas especializadas. Además, la mayoría de estos pacientes presenta importantes dificultades para realizar correctamente un estudio formal de la función respiratoria con pruebas de espirometría. Otra barrera para la atención integral de nuestros pacientes adultos con AME en nuestra área sanitaria, es que actualmente no se dispone de un acceso ágil a profesionales entrenados en la administración de las diferentes escalas motoras habitualmente empleadas en el seguimiento clínico de esta patología como son el módulo RULM (Revised Upper Limb Module), la Hammersmith Functional Motor Scale Expanded (HFMSE), y la escala MFM-32 (32-item Motor Function Measurement). Por estos motivos, y por la limitación de tiempo en la consulta ambulatoria, el protocolo para el seguimiento de los pacientes adultos con AME en nuestra consulta consta de los siguientes procesos: (1) la valoración funcional de los pacientes se realiza con la administración de las escalas Egen Klassifikation Scale version 2 (EK2) y la escala ALSFRS-r (Amyotrophic Lateral Sclerosis Functional Rating Scale - revised), (2) la valoración de la función respiratoria se cuantifica a través de la medición del peak-flow (PF, L/min) [7, 8, 9, 10], y de la SNIP (Sniff Nasal Inspiratory Pressure, cmH_2_O) [11, 12, 13, 14, 15], y (3) la valoración de la funcionalidad de los miembros superiores (mano y dedos) se valora con test de los 9 palitos (9-Hole-Peg Test, 9HPT), que es el test habitualmente empleado en la consulta de Esclerosis Múltiple para la valoración de la progresión de la discapacidad en los miembros superiores en esos pacientes [16, 17, 18]. En el 9HPT se le pide al paciente que coloque y posteriormente retire las nueve clavijas de los agujeros en la tabla, una por una, usando únicamente la mano evaluada, y se cronometra el tiempo en segundos (Fig. 1). El peak-flow nos permite la valoración de la obstrucción del árbol bronquial y de la patología restrictiva de la caja torácica, y es una medida que se aproxima a la cuantificación de la presión espiratoria máxima; en la práctica se realizan 5 mediciones y se anota tanto el valor máximo (PF máximo) como la media de las 5 mediciones (PF medio). La SNIP es una medida de la fuerza de la musculatura diafragmática e intercostal, y es una aproximación al valor de la presión inspiratoria máxima. El proceso de medición de la SNIP consiste en ajustar una sonda de medición en la fosa nasal por la que mejor inspira el paciente, y se realizan entre 10–15 inspiraciones rápidas y vigorosas, quedando reflejado en el visor la mayor de las medidas.

**Fig. 1.** **Material y métodos**. Para la valoración de la función respiratoria se han empleado las técnicas del Peak-Flow y del SNIP, y para la valoración de la funcionalidad de los miembros superiores el test de las nueve clavijas (9-hole-peg test). SNIP, sniff nasal inspiración presión.

A todos los pacientes se les extrajo una muestra plasmática (tubos con EDTA) para la determinación del nivel de los neurofilamentos de cadena ligera (NFL) y de la proteína ácida fibrilar glial (GFAP, acrónimo en inglés de Glial Fibrillary Acidic Protein). Las muestras se extrajeron en la semana previa al inicio del tratamiento con risdiplam (muestra basal) y a los 12 meses del seguimiento, y tras su centrifugación (10 minutos, 40 rpm), el plasma se conservó en alícuotas a –80 °C. La determinación de los NFL plasmáticos (pg/mL) y de la GFAP (pg/mL) se realizó en el Instituto de Investigación Biomédica de Bellvitge (IDIBELL) empleando la técnica SIMOA.

Este estudio tiene el informe favorable del Comité de Ética de la Investigación con Medicamentos de Galicia (Código de Registro: 2024/142). Los pacientes dieron su consentimiento informado para su participación en el estudio.

## 3. Análisis Estadístico

El análisis estadístico se realizó utilizando el software IBM SPSS Statistics, versión 29,0 (IBM Corporation, Armonk, NY, Estados Unidos). Se empleó la estadística no paramétrica. La comparación entre los grupos de variables relacionados (pretratamiento y postratamiento a 6 y 12 meses) se realizó con el test de Friedman, y en el caso de encontrar diferencias estadísticamente significativas, se realizaron las comparaciones entre grupos con el test de Wilcoxon. Las correlaciones se realizaron con la r de Spearman. Se consideró significación estadística con un valor de *p* < 0,05.

## 4. Resultados

Las características basales de los pacientes se exponen en la Tabla 1. El tratamiento con risdiplam fue bien tolerado, con excepción de una paciente que presentó molestias gástricas durante todo el periodo de observación (12 meses), pero no supuso el abandono de la medicación por efecto adverso.

**Tabla 1.** **Características descriptivas de los pacientes**.

	Pac 1	Pac 2	Pac 3	Pac 4	Pac 5	Pac 6	Pac 7	Pac 8
Sexo (V/M)	V	V	V	V	M	M	M	V
Edad (años)	66	41	63	41	57	51	60	53
*SMN1* deleción 5q	Si	Si	Si	Si	Si	Si	Si	Si
*SMN2* (nº copias)	4	4	3	4	3	4	4	3
Escoliosis severa	No	No	Si	Si	Si	No	No	No
Fenotipo	Sitter	Sitter	Sitter	Sitter	Sitter	Sitter	Sitter	Walker
Nusinersen previo	Si (26 meses)	Si (22 meses)	No	No	No	No	No	No

V, Varón; M, Mujer.

A nivel de las variables estudiadas no hemos hallado diferencias estadísticamente significativas a los 6 meses ni a los 12 meses del seguimiento (Tabla 2). Aunque no hubo modificaciones en las escalas de valoración funcional (EK2, ALSFRS-r), a nivel de la escala EK2 destaca que para el ítem 16 (deglución) hubo una tendencia a la mejoría a los 12 meses del seguimiento, de forma 4 pacientes disminuyeron 1 punto mientras los otros 4 permanecieron estables con una puntuación de cero (*p* = 0,06) (Fig. 2). La función respiratoria medida con el peak-flow y la SNIP se mantuvo estable a lo largo del tratamiento. Sin embargo, el PF medio en el momento basal y a los 12 meses del seguimiento mostró un incremento estadísticamente significativo (244 ± 112 vs. 259 ± 124 L/min, *p* = 0,036) (Fig. 3). El 9HPT no mostró diferencias significativas para la función de la mano dominante ni de la mano no dominante.

**Tabla 2.** **Valores de las escalas funcionales en las diferentes visitas**.

	Visita basal	Visita 6 meses	Visita 12 meses	*p*
Escala EK2	15,1 ± 7,8	15,5 ± 7,3	15,4 ± 7,5	0,895
Escala ALSFRS-r	29,6 ± 6,3	30,5 ± 7,3	30,7 ± 6,6	0,147
9-HPT [mano dominante] (segundos)	31,0 ± 3,7	30,3 ± 2,4	33,2 ± 9,7	0,957
9-HPT [mano no dominante] (segundos)	43,5 ± 10,7	44,4 ± 11,4	42,5 ± 9,8	0,846
Peak-Flow (L/min)	264 ± 118	281 ± 135	272 ± 128	0,096
SNIP (cmH_2_O)	72 ± 24	76 ± 30	73 ± 29	0,657

Las comparaciones se realizaron con el test de Friedman; EK2, Egen Klassifikation versión 2; 9-HPT, test de los 9 palitos.

**Fig. 2.** **En la figura se muestran las puntuaciones en la escala EK2 realizadas en el momento basal y a los 6 y 12 meses del seguimiento**. No se encontraron diferencias estadísticamente significativas (test de Friedman).

**Fig. 3.** **En la figura se muestran los valores del peak-flow máximo de los pacientes adultos con AME obtenidas en el momento basal, y a los 6 y 12 meses del seguimiento**. Los pacientes 3, 4 y 5 presentaban una escoliosis severa (A). Los pacientes presentaron una mejoría en el valor de peak-flow medio a los 12 meses de iniciado el tratamiento (B).

El nivel plasmático de GFAP no se modificó a los 12 meses de tratamiento con risdiplam (97 ± 55 vs. 96 ± 49 pg/mL, *p* = 0,779), y aunque hubo un descenso en la concentración de los NFL plasmáticos tampoco se alcanzó la significación estadística (11,4 ± 4,9 vs 9,4 ± 2,7 pg/mL, *p* = 0,093). Los tres pacientes con 3 copias de SMN2 tuvieron una concentración media de NFL plasmáticos (13,64 ± 4,23 pg/mL) superior a los pacientes con 4 copias de SMN2 (10,06 ± 5,24 pg/mL). Los niveles de NFL se correlacionaron negativamente con el valor del PF máximo (r = –0,74, *p* = 0,037), con el PF medio (r = –0,76, *p* = 0,028) y el SNIP (r = –0,79, *p* = 0,021); y la GFAP se correlacionó negativamente con el valor del PF medio (r = –0,71, *p* = 0,047) (Fig. 4). Los tres pacientes que presentaban una puntuación en la escala EK2 mayor a 20 puntos presentaron los niveles más elevados de GFAP (148 ± 41 pg/mL) en comparación con los pacientes que presentaban una puntuación inferior (66 ± 37 pg/mL). Una paciente refirió desde el inicio del tratamiento con risdiplam un empeoramiento en su situación clínica, reflejado por un incremento en la puntuación de la escala EK2, de forma que pasó de un valor basal de 14 a un valor de 17 a los 12 meses del seguimiento, debido fundamentalmente a una pérdida de la funcionalidad de los miembros superiores, y que se tradujo en un incremento de 6 segundos en la prueba del 9HPT. A nivel de los biomarcadores plasmáticos se comprobó un incremento de la GFAP de 35 a 80 pg/mL y de los NFL de 4 a 8 pg/mL. Esta paciente suspendió el tratamiento con risdiplam a los 12 meses de seguimiento.

**Fig. 4.** **Biomarcadores y función pulmonar**. En la imagen superior (peak-flow) y en la media (SNIP) se muestran las correlaciones negativas entre los NFL plasmáticos basales y las medidas de la función respiratoria. En la imagen inferior, los niveles plasmáticos basales de GFAP se correlacionaron negativamente con la función respiratoria (peak-flow medio).

## 5. Discusión

Risdiplam es un modificador del empalme del pre-ARNm de la proteína SMN2 de forma que desplaza dicho empalme en el gen *SMN2* para incluir el delecionado exón 7 en el transcrito de ARNm, lo que se traduce en un aumento de la expresión de la proteína SMN funcional y estable [4].

Es difícil realizar una comparación de nuestros resultados con otros estudios clínicos de pacientes adultos con AME, ya que, aunque algunos han incluido pacientes de hasta 60 años, no se ha procedido en los mismos a realizar una comparativa con los pacientes jóvenes, y además, tampoco se han empleado las mismas escalas de valoración que en nuestro estudio [19]. Lo que sí parece demostrado es que risdiplam es un tratamiento efectivo y seguro, que estabiliza o mejora a los pacientes con AME (tipo I, II y III), que su efecto es mayor en etapas presintomáticas y en estadios tempranos de la enfermedad, y se ha sugerido que puede no tener un efecto favorable en la función respiratoria lo que se atribuye a las deformidades de la caja torácica [6, 19].

En los pacientes adultos con AME la expectativa de tratar con nusinersen o con risdiplam es conseguir una ralentización de la progresión de la enfermedad y que los pacientes viven como una estabilidad en su situación clínica [20]. En este contexto, en nuestra serie de pacientes, hemos comprobado estabilidad a nivel de las escalas de valoración funcional (EK2 y ALSFRS-r) y en la destreza manual con el 9-HPT. Sin embargo, como grupo, nuestros pacientes en tratamiento con risdiplam presentaron mejorías significativas a nivel de la deglución (item 16 de la escala EK2) y de la función respiratoria (peak-flow). A nivel individual los pacientes describieron el impacto del tratamiento de una forma muy heterogénea, así unos refirieron mejorías en el control cefálico, o en la deglución, o en la sensación de llenar el pecho de aire con más facilidad, o en la facilidad para llevar el antebrazo desde la posición pendular al reposabrazos, o una mayor facilidad para flexionar el antebrazo y llevar la mano a la boca, o en tener una tos más eficaz para movilizar secreciones.

El 9HPT fue capaz de detectar un empeoramiento de la funcionalidad de los miembros superiores en dos pacientes, así el paciente 1 refirió, desde los tres meses previos a la evaluación realizada a los 12 meses, una mayor debilidad para la extensión de la muñeca (acción necesaria para realizar el 9HPT), y la paciente 6 comunicó que, a pesar del inicio del tratamiento con risdiplam, su clínica previa de pérdida progresiva de la función de los miembros superiores progresó durante los meses del tratamiento. Esta paciente es la comentada previamente en el apartado de resultados y finalmente suspendió el tratamiento con risdiplam a los 12 meses de seguimiento.

En sujetos sanos el valor de los NFL en suero es menor de 10 pg/mL, siendo el valor sérico un 25% superior a los valores plasmáticos [21, 22]. Nuestros biomarcadores están determinados en plasma, por lo que el valor estimado de NFL séricos en nuestra serie correspondería a una concentración de 14 pg/mL, indicando que los NFL de los pacientes con AME de nuestro estudio son superiores a los de controles sanos. También es conocido que los niveles de los NFL son más altos en los pacientes con AME con ≤2 copias de *SMN2* frente a los que presentan un mayor número de copias, y este dato puede influir en el valor absoluto de nuestros pacientes que presentan 3 o 4 copias y, por tanto, expresarían una menor concentración de NFL [23]. En nuestro estudio la concentración media de los biomarcadores GFAP y NFL no se modificó significativamente con el tratamiento con risdiplam, pero es de destacar que los pacientes con puntuación en la escala EK2 ≥20 presentaron las concentraciones más altas de la GFAP, sugiriendo una relación de este biomarcador con el estado funcional de los pacientes. También encontramos una correlación negativa entre los valores del peak-flow y de la SNIP con la concentración plasmática de los NFL y de la GFAP, y por tanto parecen sugerir una utilidad de estos biomarcadores en la valoración de la afectación de la función respiratoria de los pacientes con AME. No podemos descartar que a nivel individual estos biomarcadores puedan ser predictores de la evolución clínica del paciente con AME o de la respuesta al tratamiento con risdiplam. Así, en la paciente que mostró empeoramiento en la escala EK2 y en 9HPT, la concentración de ambos biomarcadores se duplicó desde el inicio del tratamiento, correlacionándose el empeoramiento clínico con el incremento en los biomarcadores plasmáticos.

Entre las limitaciones que presenta nuestro estudio se encuentran el bajo número de pacientes incluidos en el seguimiento, la falta de un grupo control, y que un 25% de la población de estudio procedía de recibir un tratamiento previo con nusinersen. Además, probablemente el tiempo de seguimiento clínico sea corto para valorar la verdadera efectividad de un tratamiento modificador de curso evolutivo de una enfermedad condicionada genéticamente.

## 6. Conclusiones

En resumen, en nuestra serie de pacientes adultos con AME de tipo IIb y III que recibieron tratamiento con risdiplam, tras un año de seguimiento, se observó una estabilización tanto a nivel clínico como bioquímico. Además, como un grupo, nuestros datos sugieren que los pacientes en tratamiento con risdiplam presentan una tendencia a la mejoría en la deglución y en la función respiratoria cuando se valora con el peak-flow. Más estudios son necesarios para confirmar la utilidad de los biomarcadores (NFL, GFAP) en el seguimiento clínico de los pacientes con AME.

## Data Availability

The datasets generated and analyzed during the current study are available from the corresponding author on reasonable request.

## References

[1] Mercuri E, Pera MC, Scoto M, Finkel R, Muntoni F (2020). Spinal muscular atrophy - insights and challenges in the treatment era. *Nature Reviews. Neurology*.

[2] Oskoui M, Servais L (2023). Spinal Muscular Atrophy.

[3] Mailman MD, Heinz JW, Papp AC, Snyder PJ, Sedra MS, Wirth B (2002). Molecular analysis of spinal muscular atrophy and modification of the phenotype by SMN2. *Genetics in Medicine: Official Journal of the American College of Medical Genetics*.

[4] Schorling DC, Pechmann A, Kirschner J (2020). Advances in Treatment of Spinal Muscular Atrophy - New Phenotypes, New Challenges, New Implications for Care. *Journal of Neuromuscular Diseases*.

[5] Maretina MA, Zheleznyakova GY, Lanko KM, Egorova AA, Baranov VS, Kiselev AV (2018). Molecular Factors Involved in Spinal Muscular Atrophy Pathways as Possible Disease-modifying Candidates. *Current Genomics*.

[6] Oskoui M, Day JW, Deconinck N, Mazzone ES, Nascimento A, Saito K (2023). Two-year efficacy and safety of risdiplam in patients with type 2 or non-ambulant type 3 spinal muscular atrophy (SMA). *Journal of Neurology*.

[7] Miquel-Gomara Perelló J, Román Rodríguez M, Grupo de Respiratorio de la Societat Balear de Medicina Familiar i Comunitaria (2002). Miquel-Gomara Perelló J, Román Rodríguez M, y Grupo de Respiratorio de la Societat Balear de Medicina Familiar i Comunitaria. Técnicas y procedimientos. Medidor de Peak-Flow: técnica de manejo y utilidad en Atención Primaria. *Medifam*.

[8] Souza CPV, Ribeiro RKC, Lima LV, Sant’Anna CC, Araújo APDQC (2018). Peak cough flow in children and young people with spinal muscular atrophy types II and III. *Fisioterapia e Pesquisa*.

[9] Veldhoen ES, Wijngaarde CA, van Eijk RPA, Asselman FL, Seddiqi N, Otto LAM (2023). Lung function decline preceding chronic respiratory failure in spinal muscular atrophy: a national prospective cohort study. *Orphanet Journal of Rare Diseases*.

[10] Veldhoen ES, Wijngaarde CA, Hulzebos EHJ, Wösten-van Asperen RM, Wadman RI, van Eijk RPA (2022). Natural history of respiratory muscle strength in spinal muscular atrophy: a prospective national cohort study. *Orphanet Journal of Rare Diseases*.

[11] Stefanutti D, Fitting JW (1999). Sniff nasal inspiratory pressure. Reference values in Caucasian children. *American Journal of Respiratory and Critical Care Medicine*.

[12] Uldry C, Fitting JW (1995). Maximal values of sniff nasal inspiratory pressure in healthy subjects. *Thorax*.

[13] Lofaso F, Nicot F, Lejaille M, Falaize L, Louis A, Clement A (2006). Sniff nasal inspiratory pressure: what is the optimal number of sniffs?. *The European Respiratory Journal*.

[14] Martínez-Llorens J, Ausín P, Roig A, Balañá A, Admetlló M, Muñoz L (2011). Nasal inspiratory pressure: an alternative for the assessment of inspiratory muscle strength?. *Archivos De Bronconeumologia*.

[15] Hart N, Polkey MI, Sharshar T, Falaize L, Fauroux B, Raphaël JC (2003). Limitations of sniff nasal pressure in patients with severe neuromuscular weakness. *Journal of Neurology, Neurosurgery, and Psychiatry*.

[16] Mathiowetz V, Volland G, Kashman N, Weber K (1985). Adult norms for the nine hole peg test of finger dexterity. *The Occupational Therapy Journal of Research*.

[17] Stam M, Wadman RI, Bartels B, Leeuw M, Westeneng HJ, Wijngaarde CA (2018). A continuous repetitive task to detect fatigability in spinal muscular atrophy. *Orphanet Journal of Rare Diseases*.

[18] Lizandra Cortés P, Poveda Verdú D, Albert Férriz A, Ñungo-Garzón NC, Domine MC, Sevilla-Mantecón T (2024). Validation of Neuromyotype: a smart keyboard for the evaluation of spinal muscular atrophy patients. *Neurologia*.

[19] Pascual-Morena C, Martínez-Vizcaíno V, Cavero-Redondo I, Martínez-García I, Moreno-Herráiz N, Álvarez-Bueno C (2024). Efficacy of risdiplam in spinal muscular atrophy: A systematic review and meta-analysis. *Pharmacotherapy*.

[20] Chabanon A, Seferian AM, Daron A, Péréon Y, Cances C, Vuillerot C (2018). Prospective and longitudinal natural history study of patients with Type 2 and 3 spinal muscular atrophy: Baseline data NatHis-SMA study. *PloS One*.

[21] Thebault S, Booth RA, Rush CA, MacLean H, Freedman MS (2021). Serum Neurofilament Light Chain Measurement in MS: Hurdles to Clinical Translation. *Frontiers in Neuroscience*.

[22] Bittner S, Oh J, Havrdová EK, Tintoré M, Zipp F (2021). The potential of serum neurofilament as biomarker for multiple sclerosis. *Brain: a Journal of Neurology*.

[23] Bayoumy S, Verberk IMW, Vermunt L, Willemse E, den Dulk B, van der Ploeg AT (2024). Neurofilament light protein as a biomarker for spinal muscular atrophy: a review and reference ranges. *Clinical Chemistry and Laboratory Medicine*.
